# *Wolbachia* and Virus Alter the Host Transcriptome at the Interface of Nucleotide Metabolism Pathways

**DOI:** 10.1128/mBio.03472-20

**Published:** 2021-02-09

**Authors:** Amelia R. I. Lindsey, Tamanash Bhattacharya, Richard W. Hardy, Irene L. G. Newton

**Affiliations:** a Department of Biology, Indiana University, Bloomington, Indiana, USA; b Department of Entomology, University of Minnesota, St. Paul, Minnesota, USA; University of Liverpool; Max Planck Institute for Marine Microbiology

**Keywords:** pathogen blocking, symbiosis, metabolism, *Drosophila melanogaster*, arbovirus, Sindbis virus, *Drosophila*, endosymbionts, host response

## Abstract

*Wolbachia* is a maternally transmitted bacterium that manipulates arthropod and nematode biology in myriad ways. The *Wolbachia* strain colonizing Drosophila melanogaster creates sperm-egg incompatibilities and protects its host against RNA viruses, making it a promising tool for vector control. Despite successful trials using *Wolbachia*-transfected mosquitoes for dengue control, knowledge of how *Wolbachia* and viruses jointly affect insect biology remains limited. Using the Drosophila melanogaster model, transcriptomics and gene expression network analyses revealed pathways with altered expression and splicing due to *Wolbachia* colonization and virus infection. Included are metabolic pathways previously unknown to be important for *Wolbachia*-host interactions. Additionally, *Wolbachia*-colonized flies exhibit a dampened transcriptomic response to virus infection, consistent with early blocking of virus replication. Finally, using *Drosophila* genetics, we show that *Wolbachia* and expression of nucleotide metabolism genes have interactive effects on virus replication. Understanding the mechanisms of pathogen blocking will contribute to the effective development of *Wolbachia*-mediated vector control programs.

## Abstract (precis)

Recently developed arbovirus control strategies leverage the symbiotic bacterium *Wolbachia*, which spreads in insect populations and blocks viruses from replicating. While this strategy has been successful, details of how this “pathogen blocking” works are limited. Here, we use a combination of virus infections, fly genetics, and transcriptomics to show that *Wolbachia* and virus interact at host nucleotide metabolism pathways.

## INTRODUCTION

*Wolbachia* is an alphaproteobacterium that establishes intracellular infections within arthropod and nematode hosts. *Wolbachia* is well characterized for inducing reproductive manipulations of arthropods in order to facilitate maternal transmission and spread throughout a population. In many cases, this reproductive manipulation is linked to the ability to protect the same host from secondary infections with pathogens, especially RNA viruses (1). The *Wolbachia* strain infecting Drosophila melanogaster (*w*Mel) both induces sperm-egg incompatibilities (known as cytoplasmic incompatibility [CI]) and blocks pathogens (2). These phenotypes have made the *w*Mel *Wolbachia* strain highly desirable for use in vector control programs. Indeed, Aedes aegypti mosquitoes transfected with the *w*Mel *Wolbachia* strain form the basis of many ongoing vector control programs aimed at reducing the impact of vector-borne diseases such as dengue and chikungunya (3–6).

Despite the utility of *Wolbachia* in controlling vector populations and vector-borne pathogens, our understanding of the *Wolbachia*-host relationship remains limited. The pathogen-blocking phenotype of *w*Mel is consistently recovered across many host species and pathogen challenges to which it has been introduced (5, 7–11). Studies point to viruses being blocked early in infection as a result of host cell physiology that has been altered by *Wolbachia*’s presence (1, 7, 12–14). However, *Wolbachia*’s effect on different hosts manifests in different ways at the cellular level, including perturbations of cholesterol availability, differential expression of host proteins, induction of the RNA interference (RNAi) pathway, and induction of immune pathways via reactive oxygen stress (12, 13, 15–18). While these differences in host cellular environment have all been implicated in pathogen blocking, none can completely explain the phenotype across host-*Wolbachia* combinations. It is easy to imagine that *Wolbachia* would have very different effects on the intracellular environment of native and nonnative hosts, which we previously reviewed in detail (1).

While it is well understood that *Wolbachia* colonization results in the differential expression of host genes, it is incredibly surprising that until now this has not been investigated in Drosophila melanogaster, comparing *Wolbachia-*colonized and *Wolbachia*-free whole animals. Previous studies have investigated (A) immune gene expression via qRT-PCR (13), (B) *Drosophila* cell lines with and without *w*Mel (7, 19), (C) whole-animal transcriptome sequencing (RNA-Seq) in other organisms (including mosquitoes [20–22], nematodes [23], leafhoppers [24], and parasitoid wasps [25]), and (D) RNA-Seq of *Drosophila* and *w*Mel across fly development (but without a comparison to flies without *Wolbachia*) (26).

Drosophila melanogaster is the native host for *w*Mel, representing a stable host-microbe relationship (27) and the organismal context in which the pathogen-blocking phenotype of *w*Mel evolved. While *Drosophila* is not a native vector for arboviruses, *Wolbachia* does significantly reduce replication of arboviruses such as Sindbis virus (SINV) in Drosophila melanogaster (13). The genetic tools available for both *Drosophila* and the type alphavirus SINV are useful for fundamental explorations of the mechanisms of intracellular infections and determinants of virus infectivity (28). Ultimately, understanding mechanisms of pathogen blocking and their evolution will facilitate the long-term success of *Wolbachia*-mediated vector control. Below we present a comprehensive RNA-Seq analysis of the effect of *Wolbachia* colonization, SINV infection, and their interactive effects in the D. melanogaster host.

## RESULTS

### *Wolbachia* colonization and virus infection globally affect fly transcription.

We used a block design (flies with or without virus, and with or without *Wolbachia*) with a time series (6, 24, and 48 h after injection with virus or saline), to assess the effect of *Wolbachia* and virus on *Drosophila* and *Wolbachia* gene expression. We generated ∼1.56 billion reads with a mean quality score of 34.21 across 48 libraries. On average, each library had 32.5 million reads. We detected no significant contamination in our libraries: libraries derived from *Wolbachia*-free flies had few reads mapping to the *Wolbachia* genome, and they were likely from the microbiome as they mapped only to conserved portions of rRNA genes and had perfect BLAST hits to genera such as *Lactobacillus* and *Acetobacter*, which are core components of the Drosophila melanogaster gut microbiome (29). Similarly, libraries derived from phosphate-buffered saline (PBS)-injected flies had a small proportion of reads map to the SINV genome, but these were only partial read matches that aligned to the SINV poly(A) tail, and not the open reading frames of the virus.

**FIG 1:**
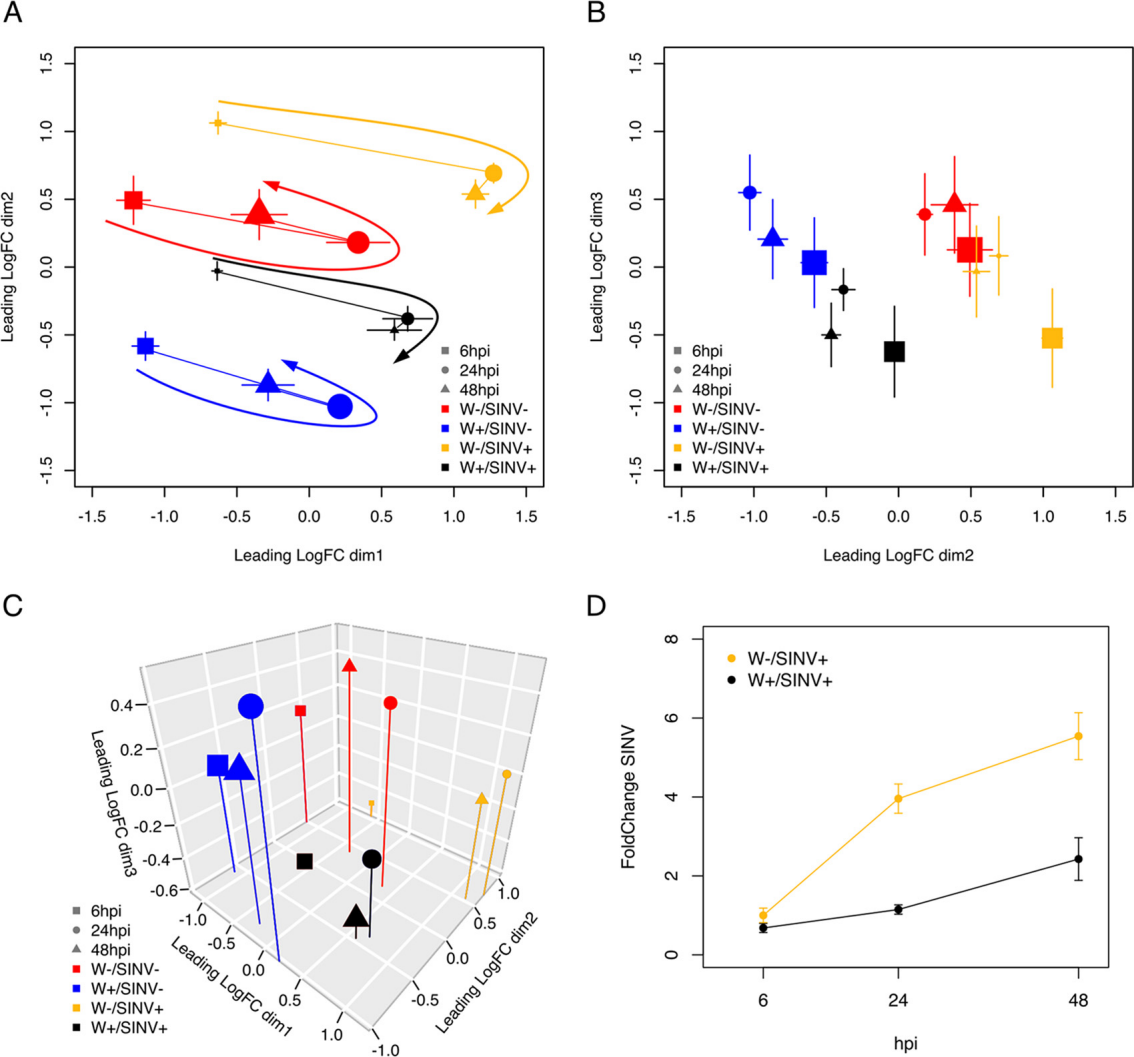
Global transcriptomic response of *Drosophila* to *Wolbachia* colonization and SINV infection. MDS plots showing similarity of total gene expression of all samples across three dimensions. Biological replicates were averaged to show their center of gravity. (A) Similarity of samples across dimensions 1 and 2. The size of points indicates how close (larger points) or far away (smaller points) they are along dimension 3, which comes in and out of the page. Lines connect time points within a *Wolbachia* W/SINV combination, and arrows show the trajectory of gene expression across time. (B) Dimensions 2 and 3 show a clustering of samples based on SINV infection and *Wolbachia* colonization. The size of points indicates how close (larger points) or far away (smaller points) they are along dimension 1, which comes in and out of the page. (C) Three-dimensional representation of the similarity of samples across all three dimensions. The size of points indicates distance from the viewer on the dimension 2 axis. In panels A and B, points are shown with ± standard error across the two dimensions shown on *x* and *y* axes. (D) Normalized abundance of SINV genomic reads across the duration of the transcriptomics experiment shows significantly reduced virus growth in the presence of *Wolbachia*.

Multidimensional scaling (MDS) plots of the global similarity in fly gene expression revealed clustering of samples based on their *Wolbachia* colonization status, SINV infection status, and time postinjection (Fig. 1A to C; also see Table S5 at Dryad [https://doi.org/10.5061/dryad.x69p8czh5]). The first dimension of the MDS plots separated samples by time, highlighted by the arrows overlaid on Fig. 1A. Indeed, flies that were injected with SINV have very different trajectories of gene expression than did the flies that were injected with PBS alone. Flies injected with PBS do show changes in gene expression across the duration of the experiment, and this is likely due to the recovery from injection, which is distinct from the changes in gene expression experienced by flies injected with SINV. The second dimension of the MDS plots primarily separated samples based on *Wolbachia* colonization, whereas dimension three separated samples based on virus infection (Fig. 1B). Three-dimensional representation of the MDS analysis showed the distinct clustering of samples based on their unique combination of *Wolbachia*-SINV-time (Fig. 1C). In contrast to the fly gene expression data, *Wolbachia* gene expression did not cluster based on virus infection status (see Fig. S1 in the supplemental material), indicating *Wolbachia* did not respond to SINV infection, as has been shown previously (7). To infer the level of pathogen blocking occurring across the duration of the transcriptomics experiment, we extracted SINV genomic reads from libraries derived from flies that received SINV injections (Fig. 1D). SINV abundance was significantly affected due to the interaction of *Wolbachia* colonization and time postinfection (generalized linear model [GLM]: χ^2^ = 11.043, df = 1, *P* = 0.0009). There were also significant effects of *Wolbachia* alone (GLM: χ^2^ = 39.877, df = 1, *P* < 0.0001) and time alone (GLM: χ^2^ = 59.751, df = 1, *P* < 0.0001). Indeed, by 48 h postinjection (hpi), for *Wolbachia*-free flies, SINV genome abundance increased 6-fold, whereas *Wolbachia*-colonized flies experienced only a 2-fold increase in SINV genome abundance (Fig. 1D). It should be noted that while *Wolbachia*-mediated pathogen blocking does occur at the level of virus genome abundance, the ultimate effects on infectivity of the virus (i.e., infectious particles) are much stronger (30).

10.1128/mBio.03472-20.1 FIG S1 Changes in gene expression. Download FIG S1, DOCX file, 0.02 MB. Copyright © 2021 Lindsey et al. 2021 Lindsey et al. This content is distributed under the terms of the Creative Commons Attribution 4.0 International license.

### *Wolbachia* colonization results in the differential expression of many cellular pathways.

Differential expression analyses revealed 237 loci that were significantly differentially expressed due to *Wolbachia* colonization, regardless of time and virus infection (Fig. S2; also see Table S2 at Dryad [https://doi.org/10.5061/dryad.x69p8czh5]). Of these, 123 were upregulated and 114 were downregulated in the *Wolbachia*-colonized flies. We also detected significant differences is isoform usage due to *Wolbachia*: 8 of the differentially expressed genes (DEGs) also displayed differential isoform usage, and an additional 48 genes displayed differential isoform usage without any significant changes in the overall level of gene expression (see Tables S2 and S6 at Dryad [https://doi.org/10.5061/dryad.x69p8czh5]). Changes in isoform usage included changes to both exon usage and/or the transcribed regions of 3′ and 5′ untranslated regions (UTRs). In total, 285 genes were either differentially expressed and/or displayed differential isoform usage.

10.1128/mBio.03472-20.2 FIG S2 Transcriptomic response to *Wolbachia* colonization. Download FIG S2, DOCX file, 1.1 MB. Copyright © 2021 Lindsey et al. 2021 Lindsey et al. This content is distributed under the terms of the Creative Commons Attribution 4.0 International license.

**FIG 2:**
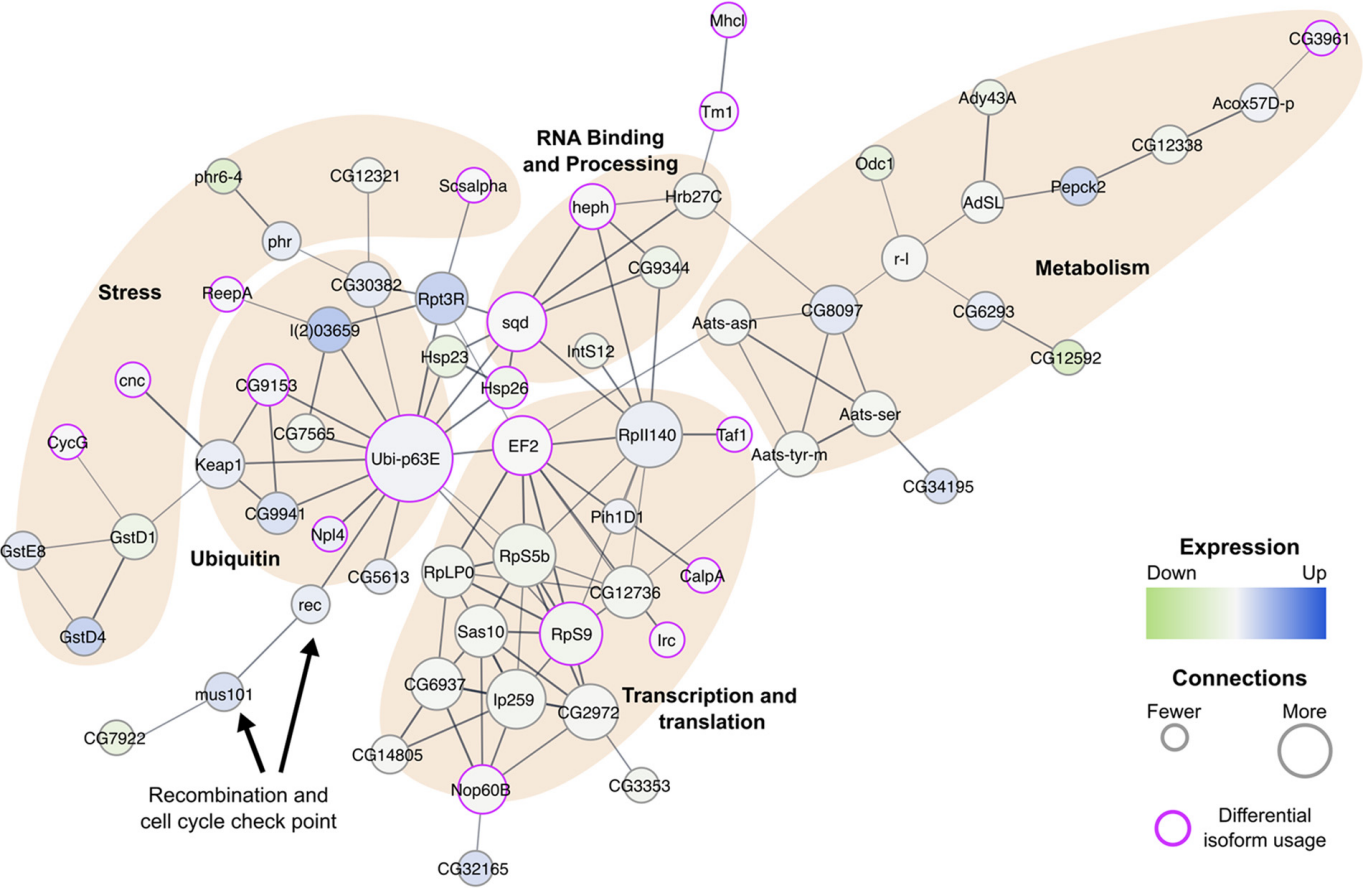
*Wolbachia-*responsive gene network comprises stress, ubiquitin, RNA binding and processing, transcription and translation, and metabolism pathways. STRING was used to identify the core network of interactions at a confidence threshold of 0.6 (relatively stringent). The size of a node corresponds to the number of connections to other nodes in the network. The color of a node corresponds to the level of expression relative to *Wolbachia*-free samples, where dark blue is upregulation of gene expression and light green is downregulation of gene expression. Purple outlines indicate significant differences in transcript usage due to *Wolbachia* colonization. The weights of edges connecting the nodes indicate confidence of the interaction. Functional categories are indicated using shaded regions.

We identified a core set of these 285 differentially expressed genes/isoforms that were predicted to interact with each other (Fig. 2). Annotation of the core network revealed distinct processes and pathways that have perturbed gene expression patterns associated with *Wolbachia* colonization. These include stress responses, ubiquitin-related processes, metabolic functions, transcription and translation, RNA binding and processing, and recombination and cell cycle checkpoint.

### Host response to virus infection varies depending on time and *Wolbachia* colonization.

**FIG 3:**
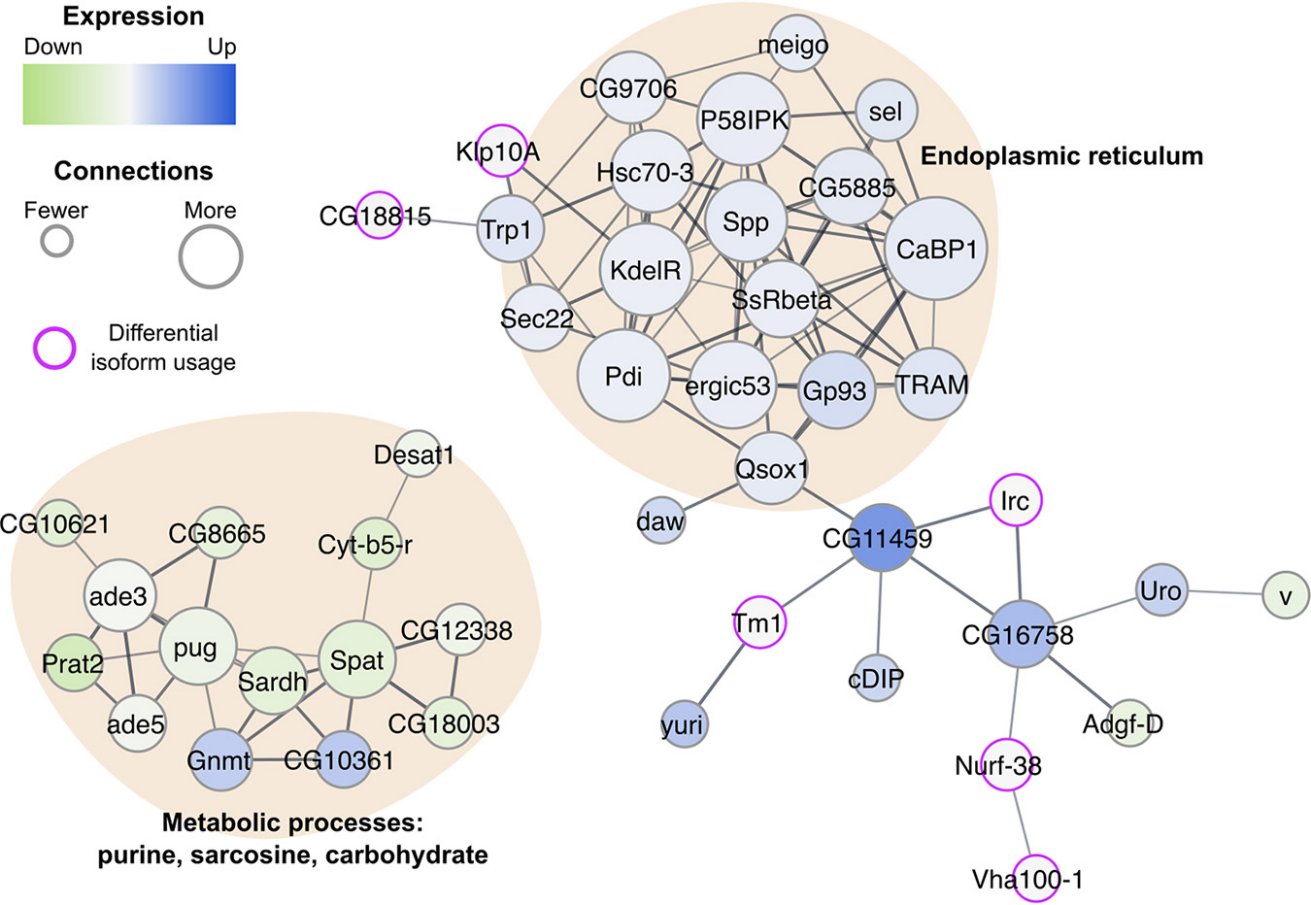
SINV-responsive core network comprises metabolic processes and endoplasmic reticulum pathways. STRING was used to identify the core network of interactions between proteins at a confidence threshold of 0.6 (relatively stringent). The size of a node corresponds to the number of connections to other nodes in the network. The color of a node corresponds to the level of expression relative to SINV-free samples, where dark blue is upregulation of gene expression and light green is downregulation of gene expression. Purple outlines indicate significant differences in transcript usage due to SINV infection. The weights of edges connecting the nodes indicate confidence of the interaction. Functional categories are indicated using shaded, annotated regions.

We identified 157 genes that were significantly differentially expressed due to virus infection (see Table S3 at Dryad [https://doi.org/10.5061/dryad.x69p8czh5]). For 15 of these genes, time also had significant interactive effect on their level of expression, which is consistent with the MDS analyses (see Table S4 at Dryad [https://doi.org/10.5061/dryad.x69p8czh5]). Virus infection resulted in significant differences in isoform usage for 38 genes, two of which were also significantly differentially expressed at the gene level (see Table S6 at Dryad [https://doi.org/10.5061/dryad.x69p8czh5]). In total, 193 genes were differentially expressed and/or displayed differential isoform usage due to SINV. Again, we clustered genes with significant differences in expression due to virus, or virus*time based on their predicted interactions and identified a core network of genes (Fig. 3). In contrast to the *Wolbachia* colonization core network, we find only two major functional categories represented in the SINV network: endoplasmic reticulum-associated processes and metabolic processes (mostly purine, sarcosine, and carbohydrate) (Fig. 3).

**FIG 4:**
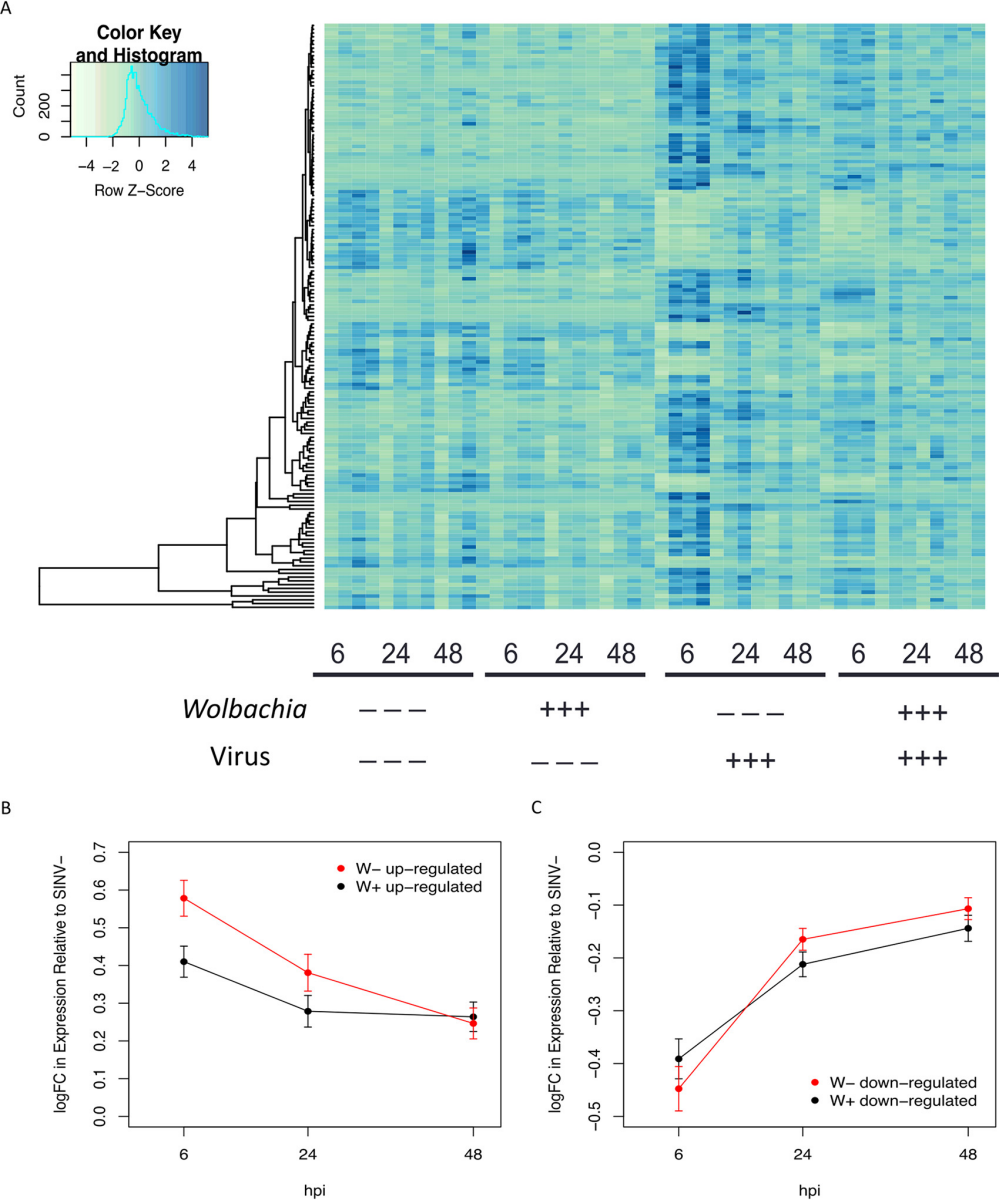
*Wolbachia* colonization alters the magnitude of response to virus infection. (A) Heatmap of the 157 genes significantly differentially expressed at the gene level, in response to SINV infection at an FDR-adjusted *P* value of 0.05 and a fold change of >2. *Wolbachia* colonization, SINV infection, and time point are indicated under each set of samples, with biological replicates adjacent to each other. (B and C) Average log(fold change [FC]) in gene expression of virus-responsive genes for W^+^ (black) and W^−^ (red) samples, relative to SINV-free flies. (B) Genes upregulated upon SINV infection. (C) Genes downregulated upon SINV infection.

While there are limited genes that had significant changes in expression due to virus*time on a per-gene basis, it is clear that global expression patterns of all the virus-responsive DEGs vary across the duration of the experiment, which is consistent with the recovery patterns identified in the MDS plots (Fig. 1). Additionally, while we did not identify any individual genes with altered expression due to the interaction of *Wolbachia* and virus, it is clear that on a global level, *Wolbachia*-free flies responded more dramatically to virus infection (Fig. 4 and Fig. S3). For DEGs that were upregulated upon virus infection, it was significantly more likely that any given upregulated DEG was more highly expressed in the *Wolbachia*-free flies than in the *Wolbachia*-colonized flies (χ^2^ = 86.26, df = 2, *P* < 0.0001). It should be noted that these differences are subtle enough on a per-gene basis that they would not meet the criteria for an interactive effect of *Wolbachia* and virus, but across the set of upregulated DEGs, we identified significant differences in the average log-fold change in gene expression between *Wolbachia*-free and *Wolbachia*-colonized flies. Upregulated DEGs were significantly more highly expressed due to the interaction of *Wolbachia* colonization and time postinfection (ANOVA: F_1,650_ = 4.687, *P* = 0.0308). There were also significant effects of *Wolbachia* alone (ANOVA: F_1,650_ = 5.668, *P* = 0.0176) and time alone (ANOVA: F_1,650_ = 29.123, *P* < 0.0001). Indeed, at 6 hpi, for *Wolbachia*-free flies, upregulated virus-responsive DEGs had an average of a 3.79-fold increase in expression relative to flies without virus, whereas *Wolbachia*-colonized flies on average experienced only a 2.57-fold increase in expression of the same DEGs (Fig. 4B and Fig. S3A). This result suggests that *Wolbachia* colonization results in a muted host response to virus infection.

10.1128/mBio.03472-20.3 FIG S3 *Wolbachia* colonization results in a muted response to virus infection. Download FIG S3, DOCX file, 1.8 MB. Copyright © 2021 Lindsey et al. 2021 Lindsey et al. This content is distributed under the terms of the Creative Commons Attribution 4.0 International license.

In contrast, the interaction between *Wolbachia* colonization and time did not affect downregulated, virus-responsive DEGs (ANOVA: F_1,272_ = 2.100, *P* = 0.1480). Additionally, *Wolbachia* colonization alone had no significant effect on the change in expression of downregulated virus-responsive DEGs (ANOVA: F_1,272_ = 0.142, *P* = 0.7070). In other words, genes that were downregulated in response to virus infection did not show a significant effect based on *Wolbachia* colonization. Time postinfection was the only factor that had a significant effect on the level of DEG expression (ANOVA: F_1,272_ = 88.345, *P* < 0.0001), as downregulated DEGs were most strongly downregulated at 6 hpi, and expression levels increased as flies recovered (Fig. 4C and Fig. S3B). However, we likely only see differences in the magnitude of response between *Wolbachia*-colonized and *Wolbachia*-free flies for upregulated DEGs and not downregulated genes due to decreased expression being bound by zero (or, no expression).

### *Wolbachia*-responsive and virus-responsive networks interact.

While we did not identify any genes with expression levels that changed due to the interaction of *Wolbachia* and virus (either including or excluding the time factor), we identified 34 genes that responded interactively to Wolbachia*virus and/or Wolbachia*virus*time at the level of isoform usage (Table 1; see also Tables S6 and S7 at Dryad [https://doi.org/10.5061/dryad.x69p8czh5]). These genes with interactive effects at the level of splicing were also significantly differentially expressed due to either *Wolbachia* or virus alone. These 34 differentially spliced genes include a range of predicted functions including transcription and translation (*eEF2*, *MED26*, and *da*), cytoskeletal organization (*sickie*, *CAP*, *Eb1*, *hts*, and *Klp10A*), nucleotide metabolic processes (*Pde11*), and immune and stress responses (*Irc* and *cert*), among others (Table 1; see also Tables S6 and S7 at Dryad [https://doi.org/10.5061/dryad.x69p8czh5]).

**TABLE 1:** Genes with isoform usage patterns that were significantly affected by the interaction of virus and *Wolbachia*

FlyBase ID	Gene	Notes	Abbreviated GO annotation^*a*^
FBgn0000559	eEF2		Translational elongation
FBgn0001225	Hsp26		Stress responses
FBgn0004509	Fur1	*Wolbachia* suppressor (40)	Neurotransmitter and protein processing
FBgn0013765	cnn	*Wolbachia* enhancer (40)	Mitotic spindle organization
FBgn0016687	Nurf-38		Chromatin remodeling, signaling
FBgn0020370	TppII		Proteolysis
FBgn0023522	CG11596		Carnosine metabolic process
FBgn0026415	Idgf4		Chitin metabolism
FBgn0027066	Eb1		Spindle organization and elongation; sensory development and locomotion
FBgn0027569	cert		Sphingolipid metabolism and transport
FBgn0030087	CG7766		Glycogen metabolism and protein phosphorylation
FBgn0030268	Klp10A		Spindle organization
FBgn0030503	Tango2		Golgi organization and protein secretion
FBgn0030504	CG2691		N/A
FBgn0032906	RPA2		DNA repair
FBgn0033504	CAP		Sensory perception
FBgn0034075	Asph	*Wolbachia* enhancer (40)	Peptidyl-aspartic acid hydroxylation
FBgn0036932	CG14184		Endomembrane system transport and localization
FBgn0037810	sle		Nucleolus organization
FBgn0037944	CG6923		Ubiquitin-dependent protein catabolic process
FBgn0038465	Irc		Oxidation-reduction process
FBgn0038470	CG18213		N/A
FBgn0038535	alt	*Wolbachia* enhancer (40)	N/A
FBgn0039350	jigr1	*Wolbachia* enhancer (40)	Regulation of gene expression
FBgn0039466	CG5521		Regulation of GTPase activity
FBgn0039923	MED26		Regulation of gene expression
FBgn0042138	CG18815		Protein depalmitoylation
FBgn0043799	CG31381		tRNA modification
FBgn0052264	CG32264		Actin cytoskeleton reorganization
FBgn0053193	sav		Signaling and growth regulation
FBgn0085370	Pde11		Signal transduction
FBgn0263391	hts		Meiotic spindle organization, actin organization
FBgn0263873	sick	Proviral (72)	Actin organization, nervous system development, response to bacterium
FBgn0267821	da		Development and cell differentiation

a Full annotations can be found in Table S7 at Dryad (https://doi.org/10.5061/dryad.x69p8czh5). N/A, not available.

**FIG 5:**
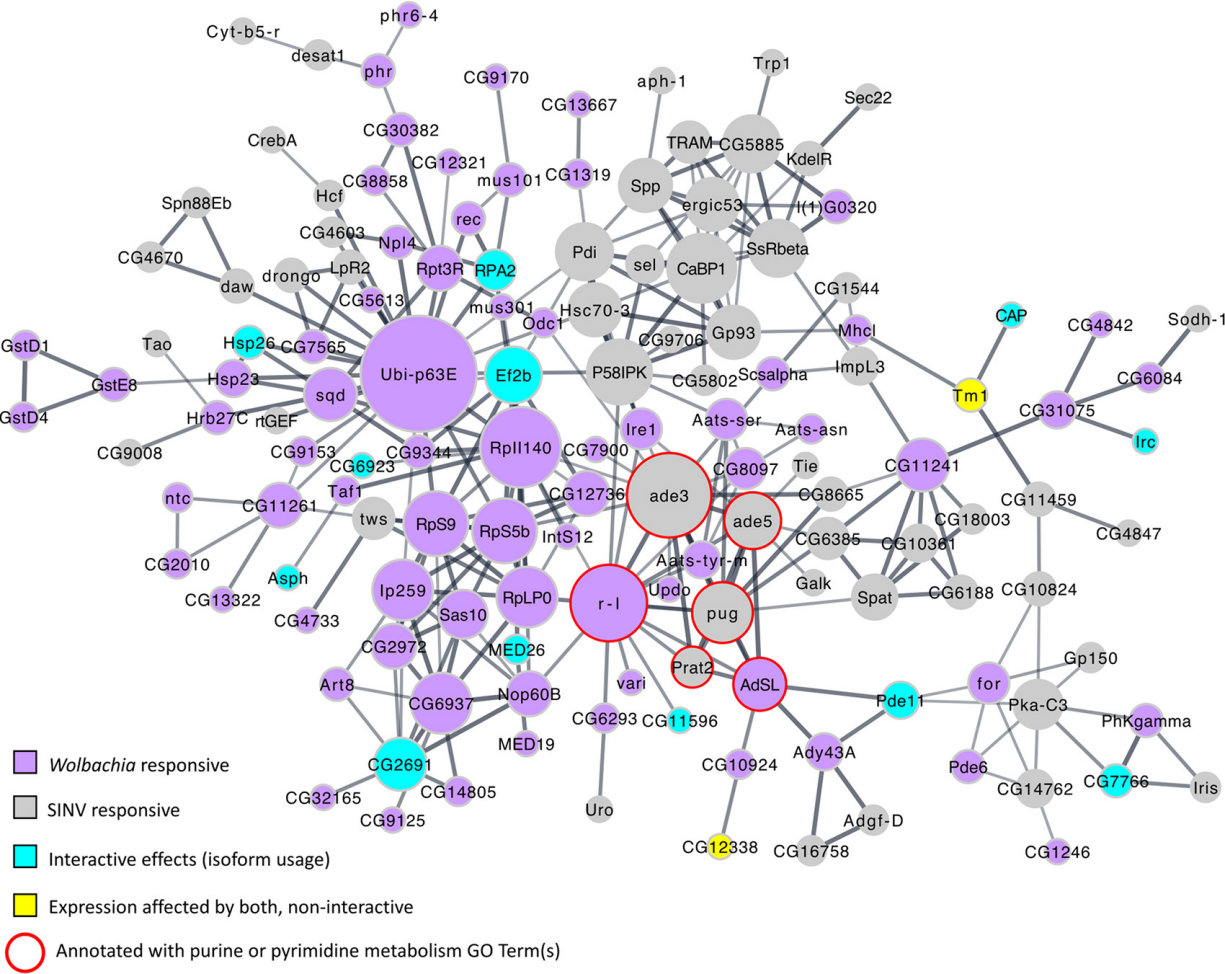
Virus- and *Wolbachia*-responsive genes are enriched for metabolic processes and interconnected around nucleotide metabolism. All infection-responsive genes were clustered to look for connectivity of the *Wolbachia*-responsive and virus-responsive gene sets. Node size indicates the number of connections to other nodes in the network. Purple nodes are *Wolbachia* responsive, and gray nodes are virus responsive. Interactive effects on expression are indicated by blue nodes. The only significant interactive effects were differential transcript usage, so all blue nodes are differentially spliced. Yellow nodes indicate genes where both SINV and *Wolbachia* had significant effects on expression but the effect was not interactive.

Next, we clustered all infection-responsive genes (at the level of either gene expression or/and isoform usage) to determine how interconnected the *Wolbachia*- and virus-responsive gene sets are. Each gene was classified as either “*Wolbachia*-responsive.” “virus-responsive.” or “interaction-responsive” (for the 34 genes mentioned above) or as those affected by both *Wolbachia* and SINV, but noninteractively (for example, differentially expressed due to *Wolbachia* colonization, and differential isoform usage due to SINV infection). We identified one core network that includes genes across all responses, with numerous connections between *Wolbachia*-responsive, virus-responsive, and interactive response genes (Fig. 5). This clustering revealed that metabolic processes are the most interconnected between the different responses, particularly *de novo* nucleotide synthesis. Indeed, we identified numerous GO processes that were significantly enriched in the joint network, all of which were metabolic in nature (see Table S8 at Dryad [https://doi.org/10.5061/dryad.x69p8czh5]). Enrichments included amino acid metabolic processes, purine biosynthesis, and other small-molecule metabolic processes.

### Nucleotide metabolism and *Wolbachia* colonization have interactive effects on virus replication.

**FIG 6:**
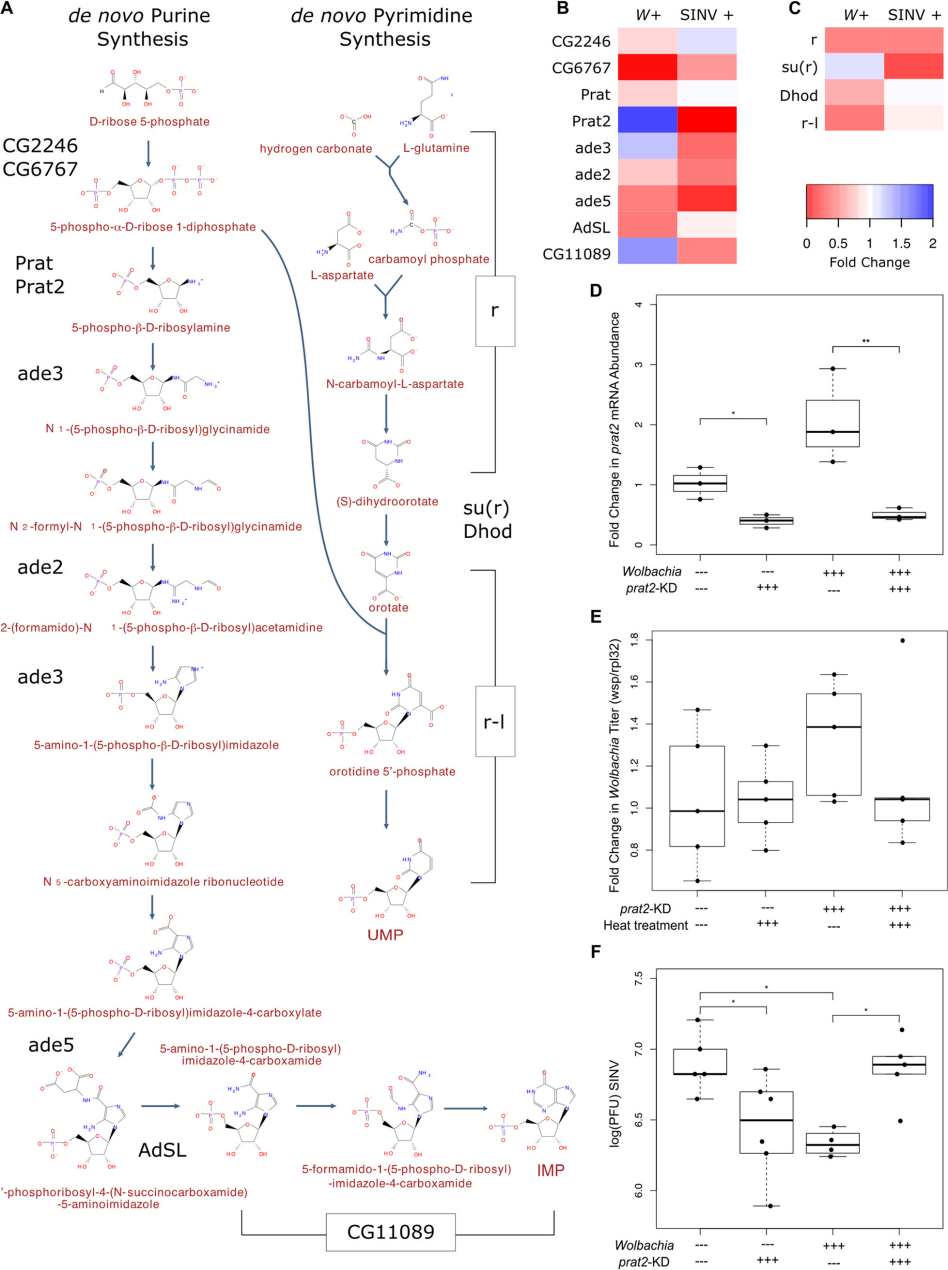
*Wolbachia* and nucleotide metabolism genes have interactive effects on virus replication. (A) *De novo* biosynthesis of purines (IMP) and pyrimidines (UMP) in Drosophila melanogaster. Genes encoding enzymes are in black. (B) Change in gene expression of *de novo* purine synthesis genes due to *Wolbachia* colonization (*W*+) and virus infection (SINV+). (C) Change in gene expression of *de novo* pyrimidine synthesis genes due to *Wolbachia* colonization (*W*+) and virus infection (SINV+). (D) *prat2* mRNA levels were quantified in flies with or without *Wolbachia* (+++ or - - -) that did or did not contain a *prat2* silencing short hairpin RNA (*prat2*-KD +++ or - - -, respectively), using qRT-PCR, relative to the expression of rpl32. Both *Wolbachia* and the presence of the shRNA resulted in significant differences in *prat2* expression (ANOVA: *Wolbachia*, F_1,8_ = 7.659, *P* = 0.0244; *prat2*-KD, F_1,8_ = 48.697, *P* = 0.0001). In sibling controls (no knockdown), *prat2* expression in *Wolbachia*-colonized flies was on average 2.06-fold higher than on *Wolbachia*-free flies. In both *Wolbachia*-colonized and *Wolbachia*-free flies, *prat2* knockdown was effective, resulting in *prat2* mRNA levels being reduced to 24.3% and 39.6% of the levels in sibling controls with the same *Wolbachia* colonization status. There was no significant difference in *prat2* mRNA levels between *Wolbachia*-colonized and *Wolbachia*-free flies with the shRNA (Tukey’s, *P* = 0.7271). (E) Flies with *Wolbachia* that did or did not contain the *prat2* targeting shRNA (*prat2*-KD +++ or - - -) were either heat shocked (10 min, 37°C) or not (heat treatment +++ or - - -) to determine if heat and/or presence of the shRNA had an effect on *Wolbachia* titer, which might affect downstream pathogen-blocking efficiency. Neither heat nor the presence of the shRNA resulted in significant differences in *Wolbachia* titer (ANOVA: *prat2*-KD, F_1,16_ = 1.985, *P* = 0.178; heat, F_1,16_ = 0.451, *P* = 0.512). (F) Flies with and without *Wolbachia* (+++ or - - -) and with or without knockdown of *prat2* (*prat2*-KD +++ or - - -) were injected with SINV to assess the effect of *Wolbachia* and *prat2* on virus replication. Viral titers from whole flies were assessed with standard plaque assays on BHK-21 cells. *Wolbachia* colonization and *prat2* knockdown had a significant interactive effect on SINV titers (ANOVA: F_1,16_ = 17.633, *P* = 0.0007). Sibling controls (no shRNA) with and without *Wolbachia* recapitulated the pathogen-blocking phenotype, with SINV titers significantly reduced, by approximately half a log, in the *Wolbachia-*colonized flies (Tukey’s, *P* = 0.0217). When *prat2* titers were knocked down, there was a *Wolbachia*-colonization-dependent effect on SINV, with knockdown being significantly proviral in the presence of *Wolbachia* (Tukey’s, *P* = 0.0350) and antiviral in the absence of *Wolbachia* (Tukey’s, *P* = 0.0469).

Given the interconnectedness of the infection-responsive networks around nucleotide metabolic processes (Fig. 5), we used fly genetics to determine if these changes in gene expression were pro- or antiviral. First, we used the RNA-Seq data to determine how *Wolbachia* colonization and virus infection affected expression of the entire *de novo* purine and pyrimidine synthesis pathways (Fig. 6A to C). These pathways are directly connected (an intermediate product of purine synthesis is required for a step of pyrimidine synthesis [Fig. 6A]), and the expression of many genes encoding enzymes involved in the pathway is significantly altered by *Wolbachia* or virus (Fig. 5). In general, the purine synthesis pathway is strongly downregulated due to virus (Fig. 6B), and the pyrimidine synthesis pathway is strongly downregulated due to *Wolbachia* [including upregulation of a suppressor, *su(r)*] (Fig. 6C). Interestingly, there are a few genes that differentially respond to *Wolbachia* and virus, such as *prat2*. *prat2* is a gene involved in the *de novo* synthesis of purine nucleotides (31, 32) and one of the most strongly downregulated in the virus-responsive gene set, expressed at <0.01% of the level of expression in PBS-injected flies (Fig. 6B). While *prat2* did not meet the threshold for statistical significance in the *Wolbachia*-responsive RNA-Seq analysis, *prat2* was upregulated in *Wolbachia*-colonized flies 1.7-fold (Fig. 6B).

Given the strong downregulation of *prat2* in virus-infected flies, the slight upregulation in *Wolbachia*-colonized flies, and the presence of *prat2* within the central “hub” of the joint network (Fig. 5), we chose *prat2* for additional analyses. We used transgenic RNAi fly lines to knock down *prat2* gene expression and assess whether or not the interaction of Prat2 and *Wolbachia* was pro- or antiviral. Knockdown was achieved using a *prat2*-targeting short-hairpin RNA (shRNA), with expression induced using heat shock conditions (Hsp.70-GAL4 driving UAS-anti-*prat2*). Sibling controls without the *prat2-*targeting shRNA recapitulated the increase in *prat2* expression seen in *Wolbachia*-colonized flies (here, ∼2-fold increase and statistically significant [ANOVA: F_1,8_ = 7.659, *P* = 0.0244]), similar to what was observed in the RNA-Seq data set with the *w*^1118^ flies (1.7-fold increase). In both *Wolbachia*-colonized and *Wolbachia*-free flies, *prat2* knockdown was effective, resulting in *prat2* mRNA levels being reduced to 24.3% and 39.6% of the sibling controls, respectively (Fig. 6D). There was no significant difference in *prat2* mRNA levels between *Wolbachia*-colonized and *Wolbachia*-free flies with the shRNA (Tukey’s, *P* = 0.7271). Neither heat shock nor knockdown had a significant effect on *Wolbachia* titer (Fig. 6E) (ANOVA: TRiP-*prat2*, F_1,16_ = 1.985, *P* = 0.178; heat, F_1,16_ = 0.451, *P* = 0.512), which is known to affect the efficiency of pathogen blocking (33, 34). Twenty-four hours postknockdown, flies were injected with SINV to determine the effect of *prat2* expression and *Wolbachia* on virus titer. *Wolbachia* colonization status and *prat2* knockdown had a significant interactive effect on SINV titers (Fig. 6F) (ANOVA: F_1,16_ = 17.633, *P* = 0.0007). Sibling controls (no shRNA) with and without *Wolbachia* recapitulated the pathogen-blocking phenotype, with SINV titers significantly reduced, by approximately half a log, in the *Wolbachia-*colonized flies (Tukey’s, *P* = 0.0217), typical of what has previously been seen in this system (13). When *prat2* expression was knocked down, there was a *Wolbachia*-colonization-dependent effect on SINV replication, with knockdown being significantly proviral in the presence of *Wolbachia* (Tukey’s, *P* = 0.0350) and antiviral in the absence of *Wolbachia* (Tukey’s, *P* = 0.0469), indicating that nucleotide metabolic processes are likely a point of interaction between host, *Wolbachia*, and virus in this system.

## DISCUSSION

*Wolbachia* colonization is well known for altering numerous physiological processes in its hosts. In many *Wolbachia*-host associations, this appears to have an effect on secondary infections, mainly with RNA viruses. Given that many different processes have been implicated in resistance to RNA viruses resulting from *Wolbachia* colonization (1), and it has been hypothesized that the preexisting state of cells with *Wolbachia* is responsible for reduced virus replication (1), we used a model system to better explore the *Wolbachia*-host relationship. We identified changes in both gene expression and isoform usage due to *Wolbachia* colonization in whole flies and identified key processes that are perturbed as a result of *Wolbachia*. This deeper look into the association allowed us to more efficiently overlay the changes that occur due to virus and identify areas of overlapping effects, regardless of whether or not they were combinatorial.

One of the major findings across our analyses is the significant amount of differential isoform usage due to *Wolbachia*, virus, and the combination of the two. The first evidence of *Wolbachia* having effects on host splicing and/or isoform usage was recently reported in a parasitoid wasp (35), and splicing is becoming increasingly appreciated as an important component of host-microbe interactions (36, 37). Whether or not *Wolbachia* directly modulates splicing via secreted factors or splicing is a host response to *Wolbachia* colonization is yet to be determined, but there are likely to be many downstream effects due to changes in isoform usage and the stoichiometry of resulting proteins.

We find that *Wolbachia* colonization affects the expression of many different biological processes, including (A) stress responses, (B) ubiquitination, (C) transcription and translation, (D) RNA binding and processing, (E) metabolism, and (F) cell cycle checkpoint and recombination. Many of these have been previously explored in host-*Wolbachia* relationships in more targeted studies (e.g., reactive oxygen species [ROS] and stress [17, 38, 39], translation [40]) and/or agree with previously identified effects that *Wolbachia* has on the host (e.g., the CI genes encode a deubiquitylase [41, 42]). The effects of *Wolbachia* on host metabolism are arguably underexplored (43), which is surprising given that *Wolbachia* must acquire all nutrients from the host, encodes a select number of its own metabolic pathways, and encodes a variety of transporters that would allow for *Wolbachia* to import specific metabolites (e.g., amino acids) (44). *Wolbachia* encodes a range of predicted amino acid importers, and it is likely that the shifts we see in the transcription of host metabolic genes are related to this. The host may be either compensating for shifts in amino acid pools due to *Wolbachia* or perhaps restricting the availability of metabolites that are available to *Wolbachia*.

We next identified the changes in gene expression and isoform usage due to the presence of virus. While the genes that responded to the viral infection were the same in flies with and without *Wolbachia*, the magnitude of response was significantly muted in flies with *Wolbachia*. This may be due to a priming effect due to *Wolbachia*’s established presence, or the virus may be so impaired from the initiation of infection that the host elicits a milder response. The virus-responsive network contained fewer cellular processes than did the *Wolbachia*-responsive network: the response to virus mainly affected the expression of endomembrane system-associated genes and metabolic pathways. Like other intracellular infections, viruses can have strong effects on host cell metabolism that may be a combination of proviral cascades initiated by the virus or antiviral responses by the host. It is notable that the virus-responsive metabolic pathways that we identify here are largely distinct from the *Wolbachia*-responsive metabolic pathways, though there is likely the potential for interaction at the level of metabolites and flux in the cell.

Proteins and metabolites directly involved in blocking need not be differentially expressed or differentially abundant to result in the decreased replication of virus. *Wolbachia*’s restructuring of the intracellular space could lead to changes in localization, posttranslational modification, or the availability of cofactors and substrates that may be critical for the expression of an antiviral effect (1). Furthermore, it is important to note that many of the processes previously identified as being involved in the blocking phenotype are (A) not mutually exclusive, (B) have the potential to act at different points in the virus life cycle, and (C) may be upstream or downstream from each other in a network of cellular changes that ultimately affect virus replication.

While it is likely that there are key differences in the mechanism(s) of pathogen blocking between different *Wolbachia*-host-virus associations, it does not exclude the possibility that there are similar upstream events (e.g., *Wolbachia* using host amino acids) that result in dissimilar downstream events that are dependent upon both (A) the host and (B) the combination of other *Wolbachia*-induced changes in physiology (e.g., a host immune response to *Wolbachia*’s presence, which is more common in nonnative *Wolbachia*-host associations [1]). In many previously published studies, a pathway has been implicated in pathogen blocking, but it was not determined how the change in host physiology occurred and whether or not that effect was directly or indirectly responsible for blocking. For example, what results in changes to expression of the Toll pathway? Antimicrobial peptides (AMPs) may be differentially expressed, but do they have a direct effect on virus replication? Do they directly interact with viral genomes and proteins? Or do the AMPs act as signaling molecules that in turn alter the expression of other host processes? Changes in lipid abundance have been associated with the antiviral effect (12, 45, 46), but it is unclear if this perturbation in lipids results in other changes to host gene expression or cellular structure, or if the virus particles themselves are unable to properly form their membrane-associated replication factories or envelopes during assembly.

Here, we identified the expression of metabolic processes as significantly altered due to both *Wolbachia* and virus. This agrees with previously published studies (43) and what we know about *Wolbachia* and virus biology. *Wolbachia* encodes a suite of amino acid importers, which likely results in altered amino acid pools in the host (44). Amino acids not only are critical for protein synthesis but also serve as precursors for many metabolic processes, including the *de novo* synthesis of purine and pyrimidine nucleotides. Here, we used fly genetics to explore the effect of *de novo* purine synthesis gene expression on the *Wolbachia*-virus-host relationship. Not only did *prat2* gene expression have an effect on viral titers, it was dependent on the presence of *Wolbachia*, which further highlights the complexity of the system and implicates multiple processes in the pathogen-blocking phenotype. In *Wolbachia*-colonized flies, where *prat2* is upregulated, *prat2* knockdown was proviral, which supports the idea of the preexisting state of *Wolbachia*-colonized flies being antiviral. It is unclear what the downstream effects of altered *prat2* expression are, and how they may be different between flies with and without *Wolbachia*. For example, knockdown of the *de novo* purine synthesis pathway may result in increased expression of the purine salvage pathway. The biochemical reactions for these different pathways have different by-products and intermediates which may have an effect on other cellular processes and virus. These downstream consequences of *prat2* knockdown may be the reason we see interactive effects of *Wolbachia* presence and *prat2* expression on virus titer. Given the differential regulation of the different metabolic pathways due to *Wolbachia* and virus (Fig. 6), there may well be other genes that have *Wolbachia*-dependent effects on the virus.

The finding that nucleotide metabolism is a source of interaction between *Wolbachia* and virus is particularly interesting given that many currently marketed antiviral drugs are known to interfere with nucleotide metabolic processes, often in the same pathways that we identify here as being perturbed due to *Wolbachia* and/or virus. For example, ribavirin and other compounds confer broad-spectrum antiviral activity by inhibition of IMP dehydrogenase, an enzyme involved in purine metabolic processes (47, 48). The antiviral activity of another compound, favipiravir, is reduced in the presence of excess purines (49). A more recently identified broad-spectrum antiviral was shown to interfere with pyrimidine metabolism via dihydroorotate dehydrogenase (50) (*dhod* in *Drosophila*), which was significantly differentially expressed due to *Wolbachia* colonization in our study. Similarly, an excess of pyrimidines rescues virus replication in the presence of this antiviral compound. A separate group of antivirals, brequinar, leflunomide, and derivatives, are also known to interfere with dihydroorotate dehydrogenase and pyrimidine pools, which is responsible for the broad-spectrum antiviral effect (51, 52).

Additional studies are needed to determine the effect of *Wolbachia* and virus on the nucleotide pools of host cells, but it is plausible that this is a major source of conflict or interaction between these two intracellular inhabitants. Indeed, metabolomic analyses, investigations in other *Wolbachia*-virus-insect systems, and mechanistic studies will likely provide a wealth of information that will help us connect transcriptomic changes to downstream events in the physiology of the host that eventually result in *Wolbachia*-mediated pathogen blocking.

## MATERIALS AND METHODS

### *Drosophila* husbandry.

A previously described line of Drosophila melanogaster, stock 6326 from the Bloomington *Drosophila* Stock Center (http://flystocks.bio.indiana.edu/), a *w*^1118^ background infected with *Wolbachia* strain *w*Mel2, and its *Wolbachia*-cleared counterpart were used in transcriptomic experiments (13). In brief, *w*^1118^ flies were cleared of their *Wolbachia* infection by three generations of tetracycline treatment. This was followed by reinoculation of the gut microbiome by transfer to bottles that previously harbored male *w*^1118^ flies that had fed and defecated on the medium for 1 week. The isogenic *w*^1118^ lines with and without *Wolbachia* were maintained in the lab separately for >20 generations prior to experimentation. *Wolbachia* colonization status was regularly confirmed using specific primers that target the *Wolbachia*-specific *wsp* locus (53). Fly stocks were maintained on standard cornmeal-agar medium at 25°C on a 24-h light/dark cycle under density-controlled conditions.

### Cell culture and virus preparation.

BHK-21 cells (American Type Culture Collection) were grown at 37°C under 5% CO_2_ in MEM (Cellgro) supplemented with 1% l-Gln, 1% antibiotic-antimycotic (Gibco), 1% nonessential amino acids, and 10% heat-inactivated fetal bovine serum (FBS) (Corning). SINV (strain TE3’2J-GFP [54]) was prepared by transfecting baby hamster kidney fibroblasts (BHK-21 cells) with 1 μg of *in vitro*-transcribed viral RNA with Lipofectamine LTX (Sigma-Aldrich) to generate a P0 virus stock, which was then used to infect new BHK-21 cells to generate P1 virus (54). The supernatant containing P1 virus was collected, purified by centrifugation over a 27% (wt/vol) sucrose cushion in 1× HNE buffer (20 mM HEPES, 0.15 M NaCl, 0.1 mM EDTA), and resuspended in 1× phosphate-buffered saline (PBS), and viral titers were determined by standard plaque assays on BHK-21 cells as done previously (54).

### *Drosophila* injections.

To determine the effect of *Wolbachia* and virus infection on fly gene expression, and the effect of virus on *Wolbachia* gene expression, we established *in vivo* systemic viral infections in adult *Drosophila*, using a block design with a time series. Flies with or without *Wolbachia* (W^+^/W^−^) were injected with either virus or saline (SINV^+^/SINV^−^) and collected at 6, 24, and 48 h postinjection (hpi). For each unique condition of W-SINV-time, we generated four biological replicates (A toD), with each replicate consisting of a pool of five virgin females. Specific conditions for generating the fly infection conditions are as follows. Five-day-old virgin female *Drosophila* flies were anesthetized with CO_2_ and injected with either (a) 50 nl sterile PBS or (b) 50 nl of freshly grown SINV (10^10^ PFU/ml in PBS) using a nanoinjector (Drummond Scientific). Pools of five flies (representing a single biological replicate) were injected in a randomized order across a 5-h time period, and capillary needles were changed between fly types (*Wolbachia* colonized or not) and injection type (PBS or SINV) to avoid cross-contamination. The exact time of injection was recorded, and the pool of five females was placed in a vial containing standard cornmeal-agar medium supplemented with antibiotic-antimycotic (Corning) and a fresh Kimwipe. Subsequently, 6-, 24-, or 48-hpi flies were flash frozen in liquid nitrogen and stored at −80°C until further processing.

### RNA extractions, library preparation, and sequencing.

RNA was extracted from pools of flash-frozen flies using TRIzol reagent (Invitrogen) following bead-beating and according to the manufacturer’s instructions. rRNAs and other uncapped RNA species were depleted from RNA samples using Terminator 5′-phosphate-dependent exonuclease (Lucigen). Following a standard phenol-chloroform-isoamyl precipitation, cDNA libraries were prepared with the NEBNext Ultra II directional RNA library prep kit (New England Biolabs) following manufacturer’s recommendations, including a 7-min fragmentation time, 10 cycles of PCR amplification, and use of a specific barcode from the NEBNext Multiplex Oligos for Illumina Index Primer Set 1 or 2 (New England Biolabs). Quality and quantity of total RNA, depleted RNA, and final libraries were assessed using a TapeStation 2200 (Agilent). Libraries were pooled in groups of 16 such that biological replicates of *Wolbachia* colonization status, SINV infection status, and time were split as evenly as possible across three runs on an Illumina NextSeq to generate 75-bp single-ended reads. Each lane contained 1 to 2 of the biological replicates for each condition. An average of 32.5 million reads was generated for each library. Further details and mapping statistics can be found in Table S1 at Dryad (https://doi.org/10.5061/dryad.x69p8czh5).

### Transcriptomic analyses.

Following demultiplexing, reads were mapped to extracted reference transcripts of either the Drosophila melanogaster reference genome (release 6.16) (55) or the *w*Mel strain *Wolbachia* genome (GenBank accession no. NC_002978.6 [44]) using the RSEM v. 1.3.0 (56) programs ‘rsem-prepare-reference’ and ‘rsem-calculate-expression’, employing the default Bowtie aligner (57). Transcript abundance was summarized and imported to R v. 3.3.1 ‘Bug in Your Hair’ (58) with tximport v. 1.2.0 (59) for use in downstream analyses. Differential gene expression and splicing were assessed with EdgeR v. 3.16.5 (60, 61), employing a TMM normalization, dispersion calculation, and a multivariate generalized linear model (‘∼*Wolbachia* * SINV * time’ for *Drosophila* expression or ‘∼SINV * time’ for *Wolbachia* expression), with quasilikelihood F tests (function ‘glmQLFit’). Splicing was assessed with the ‘diffSpliceDGE’ function using the ‘Simes’ method. Genes that were significantly up- or downregulated were defined as those with a false discovery rate (FDR) *q* value of <0.05. To check for SINV reads, libraries were mapped to the SINV TE3’2J-GFP (54) reference genome with BWA-mem2 v.2.0pre2 (62), and mapping statistics were assessed with SAMtools v.1.10 (63). To calculate SINV abundance, the poly(A) tail was masked prior to mapping.

### Nucleotide metabolism fly mutants.

Given the altered expression of genes related to nucleotide metabolism in *Wolbachia*-host and SINV-host relationships (see Results), we chose to study key fly pathways to further define these relationships. Stocks were reared and screened for *Wolbachia* using the same protocols detailed above (see “*Drosophila* husbandry”). We used a fly stock which carries a UAS-*prat2*-specific short hairpin silencing trigger (BDSC stock no. 51492, RNAi TRiP line: y^1^sc*v^1^sev^21^; P{y^+t7.7^ v^+t1.8^=TRiP.HMC03244}attP2). This stock is *Wolbachia* colonized. To generate balanced heterozygous offspring, we crossed virgin females homozygous for the hairpin to males with a third chromosome balancer (BDSC stock no.6663: w^1118^; Dr^Mio^/TM3, P{w^+mC^=GAL4-twi.G}2.3, P{UAS-2xEGFP} AH2.3, Sb^1^Ser^1^). To knock down the expression of *prat2*, the *Wolbachia*-colonized, balanced flies carrying the UAS-anti-*prat2* insert were crossed to a *Wolbachia*-free, homozygous, inducible Hsp70:Gal4-driver line (BDSC stock no. 2077: w*; P{w^+mC^=GAL4-Hsp70.PB}2). Crosses were performed in both directions to generate *Wolbachia*-free and *Wolbachia*-colonized offspring. Virgin females with and without *Wolbachia*, which contained either (i) both the Gal4-driver and the UAS-anti-*prat2* or (ii) sibling controls with the driver and the TM3 balancer, were collected and aged 4 days. At 4 days old, all flies were heat shocked at 37°C for 10 min to induce Gal4 expression. Twenty-four hours post-heat shock, flies were either injected with virus or flash frozen to assess knockdown of *prat2* or *Wolbachia* titer. For virus infections, flies were injected intrathoracically with SINV following the same injection and recovery protocol as used for setting up the transcriptomics experiment. At 48 hpi, flies were harvested and viral titers from single flies were assessed with standard plaque assays on BHK-21 cells using technical duplicates of each fly (13).

### Real-time quantitative RT-PCR analyses of *prat2* expression.

Single flies were homogenized in TRIzol reagent (Invitrogen), and RNA was extracted and DNase treated according to manufacturer’s instructions. *prat2* expression was assessed with the SensiFAST SYBR Hi-ROX One-Step kit (Bioline) according to the manufacturer’s recommendations with specific primers PP25361 (forward, 5′-GGGAATAGGACACACCCGGTA-3′; reverse, 5′-GCAGTTCACTAGCTCACCATT-3′) (64) and normalized to expression of Rpl32 (forward, 5′-CCGCTTCAAGGGACAGTATC-3′; reverse, 5′-CAATCTCCTTGCGCTTCTTG-3′ [65]) using the Livak method (66). All samples were run in technical duplicate alongside a standard curve and negative controls on an Applied Bioscience StepOnePlus qRT-PCR machine (Life Technologies).

### Real-time quantitative PCR analyses of *Wolbachia* titer.

DNA was extracted from single flies using the Qiagen DNeasy blood and tissue kit (Qiagen), according to the manufacturer’s instructions. *Wolbachia* titer was determined by amplification of the single-copy *Wolbachia* gene *wsp* and normalized to abundance of the host gene *rpl32* according to previously established protocols (65) using PowerUp SYBR Green Master Mix (ThermoFisher). All samples were run in technical duplicate alongside a standard curve and negative controls on an Applied Bioscience StepOnePlus qRT-PCR machine (Life Technologies).

### Statistics, data visualization, and network analysis.

Statistical analyses and plotting were carried out in R v. 3.3.1 ‘Bug in Your Hair’ (58). Three-dimensional plots were generated with the R package ‘plot3D’ (67), implementing the ‘scatter3D’ function. To infer pathogen blocking from the RNA-Seq data, mapped SINV read counts were normalized by library size and then to the mean read abundance of SINV at the 6-h time point for flies without *Wolbachia*. Variation in SINV abundance was assessed with a generalized linear model including *Wolbachia* colonization status, hours postinjection, and their interaction as fixed effects, and a Gaussian error distribution. Protein-protein interaction networks were constructed with STRING v.1.4.2 (68), implemented in Cytoscape v.3.6.0 (69). The confidence threshold for all networks was set to 0.600, considered stringent, so as to limit the complexity of the networks and identify the strongest interactions. Nucleotide biosynthesis pathway information was downloaded from BioCyc (70). Variation in *prat2* expression was assessed with a two-way ANOVA including *Wolbachia* colonization and *prat2* knockdown as fixed effects. Variation in *Wolbachia* titer was assessed with a two-way ANOVA including heat treatment and *prat2* knockdown as fixed effects. Variation in SINV titer was assessed with a two-way ANOVA including *Wolbachia* colonization and *prat2* knockdown as fixed effects and a log transformation of the response variable. Pairwise comparisons were conducted with the Tukey honest significant difference following all ANOVAs.

### Data availability.

The data discussed in this publication have been deposited in NCBI's Gene Expression Omnibus (71) and are accessible through GEO series accession number GSE162666 (https://www.ncbi.nlm.nih.gov/geo/query/acc.cgi?acc=GSE162666).
