# Supplementary material for: Wolbachia and Virus Alter the Host Transcriptome at the Interface of Nucleotide Metabolism Pathways
Source: mBio. 2021 Feb 9;12(1):e03472-20. doi: 10.1128/mBio.03472-20 (PMC7885120; doi:10.1128/mBio.03472-20)
Supplement: FIG S1 [file mBio.03472-20-sf001.docx]

**Figure S1. MDS plot of *Wolbachia* gene expression.** MDS plot showing similarity of total gene expression across samples. Within each SINV-timepoint combination, biological replicates were averaged to show their center of gravity +/- standard error across dimension-1 and -2. There were no significant differences in *Wolbachia* gene expression due to SINV infection. Both SINV+ and SINV- samples cluster closely together, and dimensions 1 and 2 are relatively short compared the *Drosophila* expression data.
