# Supplementary material for: Wolbachia and Virus Alter the Host Transcriptome at the Interface of Nucleotide Metabolism Pathways
Source: mBio. 2021 Feb 9;12(1):e03472-20. doi: 10.1128/mBio.03472-20 (PMC7885120; doi:10.1128/mBio.03472-20)
Supplement: FIG S2 [file mBio.03472-20-sf002.docx]

**Figure S2. Transcriptomic response to *Wolbachia* colonization**. Heatmap of the 237 genes significantly differentially expressed at the gene level, in response to *Wolbachia* colonization at an adjusted p-value of 0.05 and a fold change >2. *Wolbachia*-colonization, SINV-infection and timepoint are indicated under each set samples, with biological replicates adjacent to each other.
