# Supplementary material for: Wolbachia and Virus Alter the Host Transcriptome at the Interface of Nucleotide Metabolism Pathways
Source: mBio. 2021 Feb 9;12(1):e03472-20. doi: 10.1128/mBio.03472-20 (PMC7885120; doi:10.1128/mBio.03472-20)
Supplement: FIG S3 [file mBio.03472-20-sf003.docx]

**
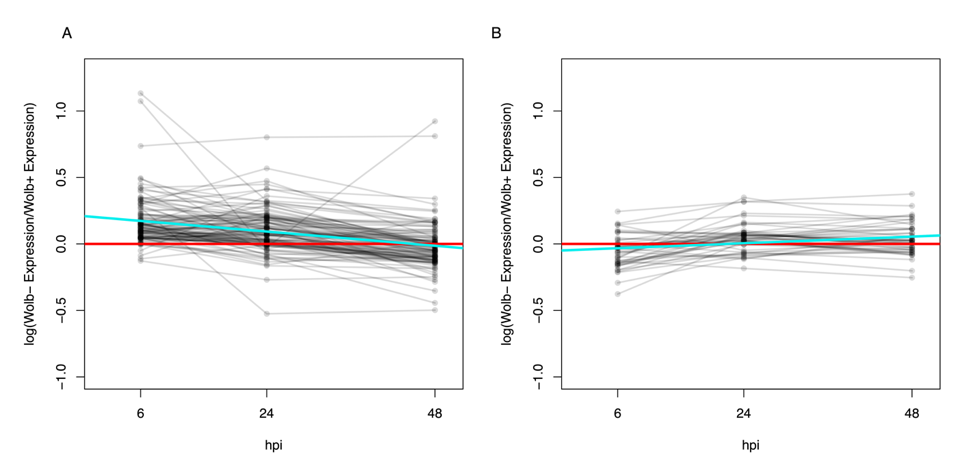
**

**Figure S3. *Wolbachia* colonization results in a muted response to virus infection.** At each time point and for each gene that was significantly differentially expressed due to SINV infection, the log of fold change in gene expression (based off TMM normalized values) was calculated for *Wolbachia*-uninfected samples injected with SINV (W-, SINV+), relative to *Wolbachia*-colonized samples injected with SINV (W+, SINV+). Values on the y-axis indicate how similar or dissimilar gene expression is, with a value of zero (red line) indicating the same level of expression in the W+ and W- samples for that gene. A larger value indicates the W- sample had higher expression for a given gene. Each grey line represents one differentially expressed “virus-responsive” gene. The blue line is a linear regression indicating whether or not the set of genes become more similar or more dissimilar in their expression between the W+ and W- samples over time. (A) Genes that are significantly upregulated in response to SINV. Positive values indicate a more exaggerated response in the W- sample. (B) Genes that are significantly downregulated in response to SINV. Negative values indicate a more exaggerated response in the W- sample.
